# *BCL-2 *(-938C > A) polymorphism is associated with breast cancer susceptibility

**DOI:** 10.1186/1471-2350-12-48

**Published:** 2011-04-01

**Authors:** Ning Zhang, Xiaoyan Li, Kai Tao, Liyu Jiang, Tingting Ma, Shi Yan, Cunzhong Yuan, Meena S Moran, Faming Liang, Bruce G Haffty, Qifeng Yang

**Affiliations:** 1 Department of Breast Surgery, Qilu Hospital, Shandong University, School of Medicine, Ji'nan, PR China; 2 Department of Obstetrics and Gynecology, Qilu Hospital, Shandong University, School of Medicine, Ji'nan, PR China; 3 Department of Therapeutic Radiology, Yale University School of Medicine, New Haven, USA; 4 Department of Statistics, Texas A&M University, College Station, USA; 5 Department of Radiation Oncology, UMDNJ-Robert Wood Johnson School of Medicine, Cancer Institute of New Jersey, New Brunswick, USA

## Abstract

**Background:**

*BCL-2 *(B-cell leukemia/lymphoma 2) gene has been demonstrated to be associated with breast cancer development and a single nucleotide polymorphism (SNP; -938C > A) has been identified recently. To investigate whether this polymorphism functions as a modifier of breast cancer development, we analyzed the distribution of genotype frequency, as well as the association of genotype with clinicopathological characteristics. Furthermore, we also studied the effects of this SNP on Bcl-2 expression *in vitro*.

**Methods:**

We genotyped the *BCL-2 *(-938C > A) in 114 patients and 107 controls, and analyzed the estrogen receptor (ER), progestogen receptor (PR), *C-erbB2 *and *Ki67 *status with immunohistochemistry (IHC). Different Bcl-2 protein levels in breast cancer cell lines were determined using western blot. Logistic regression model was applied in statistical analysis.

**Results:**

We found that homozygous AA genotype was associated with an increased risk (AA vs AC+CC) by 2.37-fold for breast cancer development and significant association was observed between nodal status and different genotypes of BCL-2 (-938C > A) (*p *= 0.014). AA genotype was more likely to develop into lobular breast cancer (*p *= 0.036). The result of western blot analysis indicated that allele A was associated with the lower level of Bcl-2 expression in breast cancer cell lines.

**Conclusions:**

AA genotype of *BCL-2 *(-938C > A) is associated with susceptibility of breast cancer, and this genotype is only associated with the nodal status and pathological diagnosis of breast cancer. The polymorphism has an effect on Bcl-2 expression but needs further investigation.

## Background

Breast cancer has become the most common female malignancy around the world. Each year, there're over one million women diagnosed with breast cancer, with approximately 400,000 deaths [1]. Like other carcinomas, breast cancer occurs based on an interaction between genetic heterogeneity and the environment. It has been reported that an accumulation of genetic variants is involved in the process of breast carcinogenesis[2]. Among these genetic variants, many of them play roles in apoptosis or cellular proliferation, since the balance between the two decides which direction to go: normal mammary development or carcinogenesis of the mammary gland [3].

Apoptosis is a highly programmed cell death, and it can be achieved by two major pathways: death-receptor pathway and mitochondrial pathway[4]. Bcl-2 family, as the most important regulator in the mitochondrial pathway, contains both anti-apoptotic proteins such as Bcl-2 and Bcl-xL and pro-apoptotic proteins such as Bax, Bad and Bak [5]. Although Bcl-2 is an oncogenic protein, the association between its expression and patient survival result is quite conflicting and seems tissue-specific. Increased Bcl-2 expression is associated with poor survival in B-cell chronic lymphocytic leukemia (CLL), prostate cancer and urinary tract transitional cell cancer [6-9]; while its high expression is associated to favorable outcome in colorectal cancer, breast cancer, non-small-cell lung cancer, renal cancer and head and neck cancer [10-15].

*BCL-2 *(B-cell leukemia/lymphoma 2) gene, located at 18q21.3 [16], consists of three exons and two promoters (P1 and P2), which have different functions. The second promoter, P2, is located 1,400 bp upstream of the translation initiation site and functions as a negative regulatory element to the P1 promoter [17,18]. Park et al. investigated the genetic variants of *BCL-2 *genes by sequencing the 24 Korean DNA samples and identified a novel single nucleotide polymorphism (SNP; -938C > A) in P2[19]. According to the findings from Nuckel et al., the -938C allele is associated with significantly increased P2 activity and binding of nuclear proteins compared with the A allele. Due to the negatively regulatory function of P2, Bcl-2 protein expression was significantly decreased in B cells derived from CLL patients carrying the -938CC genotype [20]. However, Majid et al. reported no association of Bcl-2 protein expression level with the promoter SNP or any clinical or laboratory parameters [21]. On the other hand, it has been suggested that the (-938C > A) polymorphism could serve as a survival prognosticator as well as high-risk indicator within the lymph node-negative breast cancer [22]. In order to investigate whether *BCL-2 *(-938C > A) genotype can serve as a susceptible and/or progressive factor in breast cancer, we analyzed the distribution of genotype frequency among breast cancer cases and controls, as well as the association of genotype with clinicopathological characteristics. In addition, we also chose 4 breast cancer cell lines to investigate the association between this polymorphism and Bcl-2 expression *in vitro*.

## Methods

### Patients and Samples

The study involved 114 patients diagnosed with breast cancer in Qilu Hospital (Shandong, China) between September 2008 and April 2010. All the malignant cases were classified and assessed according to the WHO classification of tumor of the breast. Among all the patients, 7 had lobular carcinoma, 87 had tubular carcinoma and 20 suffered from other malignant types. The size of the primary tumor was defined as the largest tumor diameter (cm) reported by pathologists after surgical excision. To investigate whether *BCL-2 *-938 SNP is a susceptible biomarker, 107 healthy women were involved as control group in this study. The average age of cases and controls are 49.1 ± 5.51 and 47.8 ± 11.4 years separately, and the Student's t test showed no significant difference between the two groups (*p *= 0.374). For both patient and control group, 1.5 ml whole blood sample was extracted from each participant and stored at -80°C. Written informed consent was signed by each subject and the study design was approved by the Ethical Committee of Shandong University.

### Cell culture

Breast cancer cell lines (MCF-7, MDA-MB-453, MDA-MB-468 and T-47 D cell lines) were all obtained from American Type Culture Collection (ATCC, Manassas, VA, USA), and routinely cultured in appropriate medium supplemented with 10% FBS (Haoyang, Tianjin, China), 100 U/ml penicillin, and 100 μg/ml streptomycin, under the condition of 5% CO_2 _at 37°C.

### DNA extraction

DNA from both whole blood cells and cultured breast cancer cells was extracted with TIANamp Genomic DNA Kit (Tiangen, Beijing, China), following the manufacturer's instructions. DNA concentration and purity of each sample were measured by ultraviolet spectrophotometer (GE Healthcare, Piscataway, NJ, USA). DNA samples were routinely stored at -20°C.

### PCR-restriction fragment length polymorphism analysis of BCL2 -938 polymorphism

Genotyping of the SNP -938C > A polymorphism was determined by PCR-restriction fragment length polymorphism (PCR-RFLP) method. Primers were designed according to the sequence of rs2279115 as follows: forward primer 5'-TTATCCAGCAGCTTTTCGG-3' and reverse primer 5'-GGCGGCAGATGAATTACAA-3'. In each 25 μl reaction, 1 μl genomic DNA (100 ng/μl) was amplified by 1.25 U EasyTaq DNA polymerase (Transgen, Beijing, China) with 2 μl of 2.5 mM dNTPs and 0.5 μl of each primer. The PCR conditions were set as follows: 94°C for 5 min, 35 cycles of 94°C for 30 s, 60°C for 30 s, and 72°C for 30 s and a final extension step of 72°C for 10 min.

After PCR reaction, 10 μl product from each sample was digested by BccI Enzyme (NEB, Beijing, China) for 2 hours. After electrophoresis on 3% ethidium bromide added agarose gel, photographs were taken under ultraviolet light transilluminator. To confirm our results of genotyping, several PCR products were randomly picked for sequencing.

### Immunohistochemistry (IHC) and evaluation of IHC staining

To evaluate the association between *BCL-2 *SNP-938C > A and clinicopathological parameters (estrogen receptor (ER), progestogen receptor (PR), *C-erbB2 *and *Ki67*), tissue microarrays were made and stained as described previously[23]. An already known positive case was included as the positive control, while the primary antibody was replaced with non-immune mouse serum as the negative control. Evaluation of the IHC staining results was proceeded by pathologists without knowing the Bcl-2 -938C > A genotypes. The tumor was scored positive for ER and PR when ≥10% of the tumor cells on the slide irrespective of the staining intensity. *C-erbB2 *was evaluated according to the DAKO score; complete membranous staining observed in ≥10% of tumor cells (DAKO score 2+ and 3+) was defined as *C-erbB2 *positive. More than 30% of tumor cells with the positive stained nuclear indicated a highly proliferative activity of *Ki67*.

### Western-blot analysis of Bcl-2 expression in breast cancer cell lines

Breast cancer cell lines were routinely cultured and the total protein of each cell lines was collected with RIPA lysis buffer (Sigma, St Louis, MO, USA) in the presence of protease inhibitors. Briefly, 100 μg of protein were loaded onto 12% SDS-PAGE gels and transferred to a PVDF membrane with a semi-dry blotting apparatus (Bio-Rad, Hercules, CA, USA). After blocking with 5% non-fat milk, the membrane was incubated overnight at 4°C with primary anti-Bcl-2 (1:250; Carpinteria, CA, USA), followed by binding with the secondary antibody. β-actin was used as endogenous control. The expression of each protein was quantified as the densitometry value analyzed by ImageJ. The figure shown is representative of three independent experiments.

### Statistical Analysis

The genotype and allele frequency of *BCL-2 *SNP938 were tested using the public statistical web-tool http://www.oege.org/software/hwe-mr-calc.shtml for Hardy-Weinberg equilibrium (HWE). P-value > 0.05 was considered not deviate from the equilibrium. Logistic regression model was carried out to analyze the distribution of *BCL-2 *SNP938 polymorphism between case and control group and the clinicopathological characteristics of breast cancer. All statistical tests were considered significant with a level of p ≤ 0.05. Odds ratios (OR) adjusted for age were calculated. All statistical analyses in our study were carried out with SPSS Statistics 17.0 (SPSS Inc. Chicago, Illinois, USA).

## Results

### The *BCL-2 *(-938C > A) genotype distribution

The breast cancer patients and healthy controls were all Chinese mainland women and we didn't find statistically significant difference between the two groups in the matching characteristic. Chi-square test was used to determine whether the subjects met the Hardy-Weinberg equilibrium. We confirmed that both case and control groups were compatible with the HWE, for in case and control groups the χ^2 ^value was 0.11 and 1.91 respectively, both p > 0.05.

**Figure 1:**
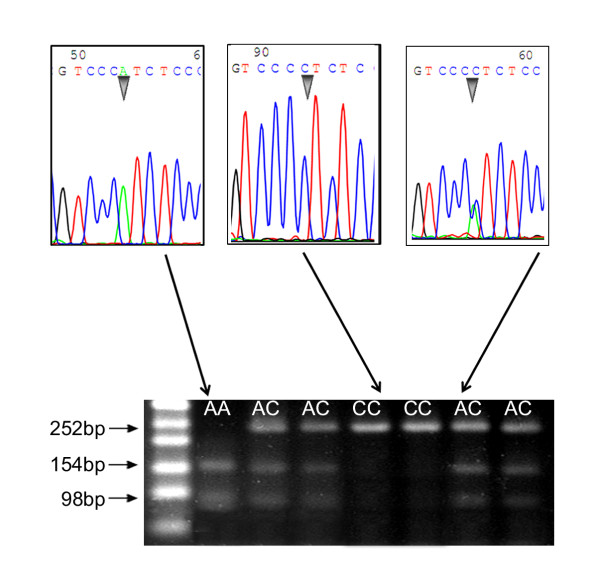
**Bcl-2 (-938C > A) genotyping results**. *BCL-2 *(-938C > A) polymorphism genotyping with PCR-RFLP and DNA sequencing. Several samples from cancer group were chosen randomly to show the PCR-RFLP results, and DNA sequencing was used to confirm our result of genotyping. Undigested 252 bp products represent CC genotype, and totally digested 154 and 98 bp products represent AA genotype; AC heterozygous genotype showed both the undigested 252 bp band and digested 154 and 98 bp bands.

As shown in Table 1, in the logistic regression model, *BCL-2 *SNP938 variant genotype CC was associated with a decreased risk for breast cancer by 0.40-fold compared with the homozygote AA. Additionally, genotype AA was associated with an increased risk for breast cancer by 2.37-fold compared with the combined genotype AC+CC. Several samples from cases were randomly chosen to show the result of genotyping (Figure 1), and the genotype distribution of *BCL-2 *SNP938 polymorphism between case and control group was shown in Table 1. Our data indicated that the homozygous AA genotype may be a susceptible genotype for breast cancer development and may increase the risk of breast cancer among Chinese women.

**Table 1:** The *BCL-2 *(-938C > A) genotype distribution in patients and controls

Genotype	No. of subjects (%)		Non-adjusted ^b^		Adjusted ^c^	
	
	Case ^a^	Control ^a^	*p *value	OR	*p *value	OR
AA	19(16.7)	8(7.5)		1.00		1.00
AC	53(46.5)	53(49.5)	0.062	0.42	0.081	0.44
CC	42(36.8)	46(43.0)	0.043	0.38	0.054	0.40
						
AC+CC	95(83.3)	99(92.5)		1.00		1.00
AA	19(16.7)	8(7.5)	0.042	2.47	0.055	2.37
						
AC+AA	72(63.2)	61(57.0)		1.00		1.00
CC	42(36.8)	46(43.0)	0.351	0.77	0.358	0.77
						
AA+CC	61(53.5)	54(50.5)		1.00		1.00
AC	53(46.5)	53(49.5)	0.651	0.89	0.711	0.90
						
All	114	107				

^a ^The χ^2 ^for HWE of case and control group is 0.11 and 1.91 respectively (both *p *> 0.05).

^b ^Logistic regression model, non-adjusted.

^c ^Logistic regression model, adjusted for diagnostic age.

All statistical tests were two-sided with a significance level of *p *≤ 0.05.

### Association between *BCL-2 *(-938C > A) polymorphism and known clinicopathological variables

Table 2 listed the association of AA and AC+CC genotypes with clinicopathological characteristics, including age at diagnosis, pathological diagnosis, tumor size and grade, nodal metastasis and status of several biological markers. The AA genotype and the occurrence of lobular breast cancer showed a strong association (*p *= 0.036). Furthermore, the association of different genotypes with the lymph nodal status was significant (*p *= 0.014). However, we found no association of the polymorphism with age at diagnosis, tumor size and grade. Regarding to the biological markers, which can predict disease prognosis and treatment outcomes, the genotypes were similarly distributed irrespective of the status of ER, PR, erbB2 and *Ki67*, neither do the receptor status or triple-negative breast cancer. Thus, the *BCL-2 *(-938C > A) polymorphism may be an indicator of lymph nodal metastasis in female breast cancer.

**Table 2:** Relationship between *BCL-2 *(-938C > A) polymorphism and known clinicopathological variables

Clinicopathological Variables	All	Genotype (%)		Adjusted ^a^	
		
		AA	AC+CC	*p *value	OR
Age					
≤ 40	21(18.9)	3(14.3)	18(85.7)		1.00
> 40	90(81.1)	15(16.7)	75(83.3)	0.790	1.20
Pathological diagnosis					
Ductal	87(76.3)	14(16.1)	73(83.9)		1.00
Lobular	7(6.1)	3(42.9)	4(57.1)	0.036	6.42
Others	20(17.6)	2(10.0)	18(90.0)	0.605	0.66
Tumor size(cm)					
≤ 2	50(53.2)	9(18.0)	41(82.0)		1.00
> 2	44(46.8)	9(20.4)	35(79.6)	0.704	1.23
Grade					
I, II	59(79.7)	10(16.9)	49(83.1)		1.00
III	15(20.3)	2(13.3)	13(86.7)	0.725	0.74
Positive lymph node					
0	34(44.7)	3(8.8)	31(91.2)		1.00
1-3	25(32.9)	3(12.0)	22(88.0)	0.672	1.44
≥ 4	17(22.4)	7(41.2)	10(58.8)	0.014	6.95
ER					
Negative	28(26.9)	6(21.4)	22(78.6)		1.00
Positive	76(73.1)	12(15.8)	64(84.2)	0.493	0.68
PR					
Negative	37(35.9)	8(21.6)	29(78.4)		1.00
Positive	66(64.1)	10(15.2)	56(84.8)	0.688	0.80
Receptor status					
Negative	26(25.2)	6(23.1)	20(76.9)		1.00
Positive	77(74.8)	12(15.6)	65(84.4)	0.115	3.06
*c-erbB2*					
Negative	73(70.2)	13(17.8)	60(82.2)		1.00
Positive	31(29.8)	5(16.1)	26(83.9)	0.936	1.05
Triple-negative					
No	87(84.5)	14(16.1)	73(83.9)		1.00
Yes	16(15.5)	4(25.0)	12(75.0)	0.444	1.67
*Ki67*					
Negative	59(58.4)	10(16.9)	49(83.1)	0.875	1.00
Positive	42(41.6)	7(16.7)	35(83.3)		1.09

^a^Logistic regression model adjusted for diagnostic age.

All statistical tests were two-sided with a significance level of *p *≤ 0.05.

### Association between *BCL-2 *(-938C > A) polymorphism and Bcl-2 expression *in vitro*

**Figure 2:**
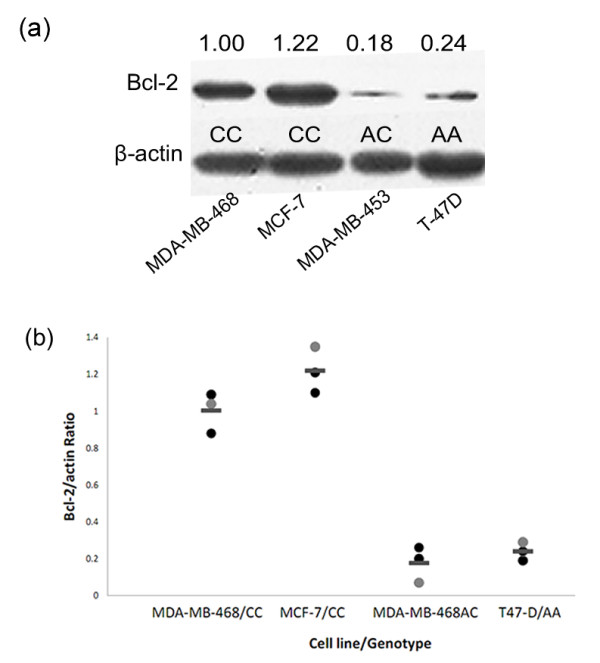
**Genotype-dependent expression of Bcl-2 protein in breast cancer cell lines**. (a) Effect of *BCL-2 *(-938C > A) polymorphism on Bcl-2 expression level in breast cancer cell lines. β-actin was used as an internal control and the expression of Bcl-2 protein was quantified as densitometry value analyzed by ImageJ. The figure shown is representative of three independent experiments. (b) Quantitative analysis of protein expression shown in the figure. Densitometry was performed using ImageJ statistical analysis. Statistical analysis was performed using the Student's t test. Horizontal bars represent the mean value of the three values of each cell line/genotype.

We further investigated the possible linkage of *BCL-2 *(-938C > A) polymorphism with Bcl-2 expression level *in vitro*; we tested the genotypes of the polymorphism and Bcl-2 expression in 4 breast cancer cell lines. Figure 2 showed the result of genotyping and western blot of the 4 breast cancer cell lines (MCF-7, MDA-MB-453, MDA-MB-468 and T-47D). From the result, we can identify an association that CC genotype correlated with high Bcl-2 expression and AA genotype displayed an intermediate level, while AC genotype correlated with the lowest expression. The ratio of Bcl-2/actin was almost 5-fold higher comparing CC genotype (1.00 ± 0.11) with AC genotype (0.18 ± 0.10; *p *= 0.0006; Student's t test), whereas no significant effect was found when comparing AA genotype (0.24 ± 0.05) to AC genotype (0.18 ± 0.10; *p *= 0.372; Student's t test). Overall, *BCL-2 *(-938C > A) polymorphism showed a contribution to Bcl-2 expression, namely allele A was associated with lower level of Bcl-2 expression and vice versa.

## Discussion

Anti-apoptotic *BCL-2 *gene plays an important role in regulation of apoptosis and triggering cell cycle arrest. Due to its role in CLL development, many studies which focused on its function and variants were carried out in leukemia previously. The *BCL-2 *(-938C > A) polymorphism, which was first identified by Park and colleagues[19], has attracted a lot of attention recently; the result of these studies[20,21,24-26] in CLL patients appeared in great controversial and no agreement has been reached. However, studies carried out in solid tumors showed a more consistent result [14,15,22,27,28], indicating *BCL-2 *(-938C > A) polymorphism may function in a tissue specific way. Since Bcl-2 protein has been reported to contribute much to breast malignancy development (or progression), we conducted this study to investigate whether this polymorphism functioned as a modifier of breast cancer development.

In our study, we found that homozygous AA genotype may be a susceptible genotype for breast cancer development and this genotype is associated with the pathological diagnosis and nodal status of breast cancer. In addition, we investigated the effect of this polymorphism on Bcl-2 expression *in vitro *and found that allele A was associated with the lower level of Bcl-2 expression.

As indicated in previous literatures, this polymorphism was located in the second promoter of the *BCL-2 *gene, which worked as a negative modulator on the first promoter and subsequently, *BCL-2 *gene expression [18]. According to the study of Nuckel et al., the presence of AA genotype was associated with decreased activity of P2 and increased Bcl-2 protein expression and was demonstrated as an unfavorable genetic marker in patients with B-CLL [20]. In contrast, we showed that allele A was associated with low Bcl-2 expression in breast cancer cell lines and AA genotype is in positive relation to breast cancer susceptibility. This result can partly explain the reason why high Bcl-2 expression is associated with better prognosis of breast cancer [12,29,30]. The different results in CLL and breast cancer may be determined by the balance between the dual function of Bcl-2 protein[31]. On one hand it plays an important role as an oncogene to inhibit apoptosis; on the other hand, it can initiate cell cycle arrest at G0 stage [32] and cause growth inhibitory effects similar to those of p53 [33]. Nevertheless, a recent study by Bachmann et al. reported no significant association was observed between Bcl-2 expression status and *BCL-2 *(-938C > A) polymorphism (*p *= 0.485) [22]. Therefore, the association of *BCL-2 *(-938C > A) polymorphism with Bcl-2 expression in primary tumor should be studied in a larger sample size and the involved molecular mechanisms need further investigation. Additionally, it was reported that Bcl-2 high expression contributed to a series of cytotoxic drugs resistance [34]. Thus, we can also investigate whether the *BCL-2 *(-938C > A) polymorphism has an effect on chemotherapy resistance in the future work.

## Conclusions

In summary, we can conclude that AA genotype of *BCL-2 *(-938C > A) may be one susceptible genotype for breast cancer, and this genotype is associated with the nodal status of breast cancer. Since Bcl-2 protein has been demonstrated to be correlated with treatment outcomes and prognosis of breast cancer, it is of great significance to find the function pattern of this polymorphism and the possible association with Bcl-2 expression. Further research is necessary to elucidate whether *BCL-2 *(-938C > A) polymorphism can serve as a prognostic biomarker for breast cancer patients.

## Competing interests

The authors declare that they have no competing interests.

## Authors' contributions

NZ, XL, KT did the genotype analysis and IHC staining. LJ, TM, SY, CY collected all the clinical samples. FL did the statistical analyses. MM, BH, QY designed the study. All authors read and approved the final manuscript

## Pre-publication history

The pre-publication history for this paper can be accessed here:

http://www.biomedcentral.com/1471-2350/12/48/prepub
